# DOES EVOLUTIONARY BIOLOGY HELP THE UNDERSTANDING OF METABOLIC SURGERY? A FOCUSED REVIEW

**DOI:** 10.1590/0102-672020190001e1503

**Published:** 2020-07-08

**Authors:** Sergio SANTORO, Caio G G AQUINO, Filippe Camarotto MOTA, Roberto Ferreira ARTONI

**Affiliations:** 1 Department of Surgery, Albert Einstein Hospital, São Paulo, SP, Brazil; 2 Department of Surgery, School of Medicine, University of São Paulo, São Paulo, SP, Brazil; 3 Evolutionary Genetics Laboratory, Department of Structural, Molecular and Genetic Biology, Ponta Grossa State University, PR, Brazil

**Keywords:** Obesity, Metabolic syndrome, Gastric bypass, Glucagon-like peptide 1, Gastric inhibitory polypeptide, Obesidade, Síndrome metabólica, Derivação gástrica, Receptor do peptídeo semelhante ao glucagon 1, Peptídeo inibidor gástrico

## Abstract

**Introduction:**

The wide net of physiological issues involved in metabolic surgery is extremely complex. Nonetheless, compared anatomy and phisiology can provide good clues of how digestive tracts are shaped for more or less caloric food, for more or less fiber, for abundance and for scarcity.

**Objective:**

To review data from Compared Anatomy and Physiology, and in the Evolutionary Sciences that could help in the better comprehension of the metabolic surgery.

**Method:**

A focused review of the literature selecting information from these three fields of knowledge in databases: Cochrane Library, Medline and SciELO, articles and book chapters in English and Portuguese, between 1955 and 2019, using the headings “GIP, GLP-1, PYY, type 2 diabetes, vertebrates digestive system, hominid evolution, obesity, bariatric surgery “.

**Results:**

The digestive tract of superior animals shows highly specialized organs to digest and absorb specific diets. In spite of the wide variations of digestive systems, some general rules are observed. The proximal part of the digestive tract, facing the scarcity of sugars, is basically dedicated to generate sugar from different substrates (gluconeogenesis). Basic proximal gut tasks are to proportionally input free sugars, insulin, other fuels and to generate anabolic elements to the blood, some of them obesogenic. To limit the ingestion by satiety, by gastric emptying diminution and to limit the excessive elevation of major fuels (sugar and fat) in the blood are mostly the metabolict asks of the distal gut. A rapid and profound change in human diet composition added large amounts of high glycemic index foods. They seem to have caused an enhancement in the endocrine and metabolic activities of the proximal gut and a reduction in these activities of the distal gut. The most efficient models of metabolic surgery indeed make adjustments in this proximal/distal balance in the gut metabolic activities.

**Conclusion:**

Metabolic surgery works basically by making adjustments to the proximal and distal gut metabolic activities that resemble the action of natural selection in the development the digestive systems of superior animals.

## Graphical Abstract

[Fig f1a]

**Figure f1a:**
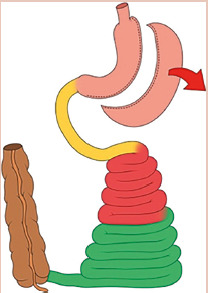
Vertical gastrectomy and different bowel functions

## RESUMO

**Introdução:**

A rede de questões fisiológicas envolvidas na cirurgia metabólica é muito complexa. No entanto, a anatomia e fisiologia comparadas podem fornecer boas pistas sobre como o trato digestivo é moldado para alimentos mais ou menos calóricos, para mais ou menos fibras, para abundância e escassez.

**Objetivo:**

Selecionar e analisar dados de Ciências Evolucionárias e Anatomia e Fisiologia Comparadas que ajudam na compreensão da cirurgia metabólica.

**Método:**

Revisão focada da literatura, selecionando informações desses três campos de conhecimento em bancos de dados da Cochrane Library, Medline e SciELO, artigos e capítulos de livros em inglês e português, entre 1950 e 2019, usando como descritores “GIP, GLP-1, PYY, type 2 diabetes, vertebrates digestive system, hominid evolution, obesity, bariatric surgery”.

**Resultado:**

O trato digestivo de animais superiores mostra órgãos altamente especializados para digerir e absorver dietas específicas..A parte proximal, diante da escassez de açúcares, é basicamente dedicada à geração de açúcar a partir de diferentes substratos (gliconeogênese). As tarefas básicas do intestino proximal consistem em fornecer proporcionalmente açúcares livres, insulina e outros combustíveis e gerar elementos anabólicos no sangue, alguns deles obesogênicos. Limitar a ingestão pela saciedade, por diminuir o esvaziamento gástrico e limitar a elevação excessiva dos principais combustíveis (açúcar e gordura) no sangue são principalmente as tarefas metabólicas do intestino distal. Mudança rápida e profunda na composição da dieta humana causa elevação nas atividades endócrinas e metabólicas do intestino proximal e redução no intestino distal. Os modelos mais eficientes de cirurgia metabólica fazem ajustes nesse equilíbrio proximal-distal das atividades metabólicas intestinais.

**Conclusão:**

A cirurgia metabólica funciona basicamente fazendo ajustes nas atividades metabólicas do intestino proximal e distal que se assemelham à ação da seleção natural no desenvolvimento dos sistemas digestivos de animais superiores.

## Graphical Abstract

[Fig f1as]

**Figure f1as:**
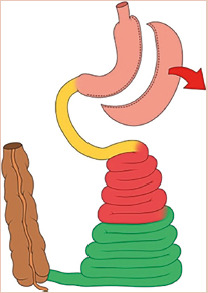
A gastrectomia vertical e as funções diferentes do intestino

## INTRODUCTION

If you want to build an efficient wing, you may go deep into aeronautical engineering and into physics, or you can copy it from a bird. The wide and deep net of physiological issues involved in metabolic surgery is extremely complex. Nonetheless, comparative anatomy can give us good clues regarding how digestive tracts are shaped for greater or lesser caloric food, for more or less fiber, and for abundance and scarcity.

In a specialized ambulatory for short bowel syndrome, some unexpected physiological observations were made in the 1990s.

It was well known that those who lost all the distal part of the small bowel would suffer more and depend more on medical support compared to extensive proximal losses. However, in some rare cases, something new appeared, i.e. for some unknown reason, some individuals who lost large amounts of a previously normal proximal part of the small bowel recovered better, as predicted, but paradoxically, many became better than they were before the loss.

How can someone loose a normal part of the body and instead of getting sick and having poorer health, become healthier and better? This observation has occurred a few times, always bringing back this intriguing question.

There was a small group of patients who, before the condition (or accident) that led them to have extensive proximal bowel resectioning, were obese, presenting with high blood pressure, high blood lipids and insulin resistance. After the adaptation that follows extensive proximal bowel resectioning (almost the whole jejunum, in these few cases), no significant malabsorption was present; the individuals were healthy and, surprisingly, not obese, not hypertensive and with normal blood sugar and lipids. How did it happen, if malabsorption were not present?

In modern times, we have clues to answer this question. It has been shown that proximal, but not distal, bowel resectioning may have a positive impact on metabolic syndrome and insulin resistance^23^, and many interesting hypotheses and physiological mechanisms are candidates to explain this phenomenon.

Evolutionary sciences may provide a potential pathway to solving this puzzle. The occurrence of random changes in living beings may, in certain circumstances, be beneficial. This is the cornerstone of evolutionary sciences. In normal conditions, mutations in the genetic code are the reasons for the modifications, and if beneficial, these changes may cause a positive selection of the evolved genes.

However, with the “recent” development of surgery, we can modify bodies without any genetic change: a surgical modification. In most situations, surgery is performed to remove or treat an ill organ. The adaptation after surgery can lead to sequelae or to a full recovery, with a patient that is as good as before. Nonetheless, as rare as a beneficial genetic mutation, surgery can produce a better being, more adapted to the current circumstances.

Based on the Compared Anatomy and Physiology, and in the Evolutionary Sciences the objective of this review was to find links with these issues and the metabolic surgery.

## METHOD

A focused review of the literature selecting information from these three fields of knowledge was done in Cochrane Library, Medline and SciELO, articles and book chapters in English and Portuguese, between 1955 and 2019, using the headings “GIP, GLP-1, PYY, type 2 diabetes, vertebrates digestive system, hominid evolution, obesity, bariatric surgery “.

## RESULTS

### Metabolic responses

#### Lessons from exclusive carnivores: the cat

Exclusive carnivores, such as the cat, in a period of scarcity and hunger keep their blood glucose at normal levels due to acontinuous endogenous production of glucose, both fromglycogenolysis(consuming glycogen reserves) orgluconeogenesis by using other possible precursors of glucose, mainly glucogenic amino acids, pyruvate, lactate and glycerol (from triglycerides). The secretion of the pancreatic hormone glucagon is mainly responsible for this endogenous production of glucose^22^.

Simultaneously, very low amounts of insulin are in circulation. The low levels of this hormone impede the consumption of glucose by body tissues. As some tissues have the capacity to consume glucose even in the absence of insulin, endogenous glucose production is basically devoted to insulin-independent tissues, mainly the central nervous system.

In this period of starvation, the muscles and other body tissues that have no access to glucose (because of the low insulin levels) consume mostly fat. Low blood insulin induces triglycerides to be broken into glycerol and free fatty acids (FFA). Glycerol can be a substrate for gluconeogenesis, thus generating glucose, as mentioned above, but FFA cannot and will generate ketone bodies that serve as fuel to the cells during the starvation.

Fat is stored in times of abundance, under higher insulin levels. Adipose tissues under very low insulin levels do the opposite process, lipolysis, to release FFA into the blood.

Animal tissues, which comprise the diet of the carnivore, are almost all protein and fat, with insignificant amounts of sugar. After a meal, an insulin response will be triggered and, even with no sugar ingestion, no hypoglycemia will occur.

The main responsible for the phenomenon is a gut hormone, glucose-independent insulinotropic polypeptide, or simply GIP. GIP is secreted after meals. Nutrients stimulate K cells in the proximal gut and provoke GIP secretion. If the glycemia is not high, GIP induces insulin secretion and a powerful glucagon secretion (which was already high due to the fasting period). Therefore, more endogenous glucose is produced - now under high insulin -, and all insulin-dependent tissues will have access to the precious glucose^6^.

This situation is a glucose feast to cells, but note that this glucose was not ingested, but produced. The enterohormone GIP has a great responsibility in this aspect. Therefore, it is not a surprise that GIP is produced throughout the small bowel and is the main incretin of exclusive carnivores, such as felines. The glucose found in the blood of a carnivore is a product of gluconeogenesis.

Cats also have L cells in the ileum like other mammals. Nutrients in the distal small bowel are a signal that the meal was significant, and GLP-1 production helps to create satiety, to block gastric emptying and, even at a certain point, to block glucagon secretion to avoid hyperglycemia after much glucose has been produced (even if none was consumed). These elements together will induce the end of a meal. It is important to highlight that even though they eat largely protein and fat, cats may present with type 2 diabetes mellitus (T2DM)^32^ and that GLP-1 analogs such as exanatide can be used to treat them, as in humans.

The important message is that most glucose is a product of gluconeogenesis and that the proximal bowel is the main actor in the process.

#### Lessons from ruminant herbivores: the cow

Ruminants are herbivorous mammals (cattle, goats, giraffes, deer, etc.) that present a “complex stomach”, usually with four chambers: rumen, reticulum, omasum and abomasum. They eat very poor forage. There is very little free sugar in these vegetables, which contain tough fiber with a large amount cellulose, hemicellulose and some protein.

The life strategy of a ruminant is rather eccentric: they eat poor food that is easy to find and that is not much disputed by other animals so that ruminants do not expend too much energy to fight for it; however, they have the hard task of taking nutrients from this feed. Mammals do not have cellulase, the enzyme that allows digestion of cellulose. Ruminants eat very large volumes and send their food to the rumen (food may return to the mouth to be chewed again - this is what“ruminare” means). In the rumen, bacteria and yeasts ferment the cellulose, generating volatile fatty acids such as acetic acid, propionic acid and other substances that can be transformed into glucose through gluconeogenesis^9^.

Curiously, bacteria retain a great part of the energy and protein from the food they digest. However, these bacteria are further digested in the abomasum. Therefore, it is correct to say that ruminants live from the nutrients they extract from forage with the help of other organisms and from the nutrients that they further obtain by digesting the organisms that helped them.

The important aspect for the spirit of this article is that, even in a ruminant herbivore, blood glucose comes mostly fromgluconeogenesis, and glucose generation is important. Ruminants do not eat significant amounts of free sugars. The proximal gut is deeply involved in the production of glucose^2^.

#### Lessons from non-ruminant herbivores: the horse

Horses expend more energy moving over longer distances and looking for food that is slightly richer; in addition to grass and hay, seeds, apples and carrots may be a meal. Horses therefore, eat a little richer food compared to a cow. Probably, this is the reason why the horse stomach is smaller than the cow’s, and has only one chamber that produces acid and pepsin. Acid interrupts fermentation, as it is unfavorable for the growth of bacteria. Enzymatic and hydrolytic digestion occurs before the fermentation of fiber.

Richer food, which costs more effort to obtain, will not be shared soon with the gut bacteria. The stomach initiates digestion, and in the small intestine, proteins and sugars are digested and absorbed before “the sharing”. A large amount of fiber with cellulose remains. The horse has a very large cecum^30^ (where the chyme enters and exits through the same aperture) and where there is a large number of bacteria. Fermentation happens intensively, generating volatile fatty acids that will serve as substrate for gluconeogenesis. Again, gluconeogenesis is a crucial source of glucose for the blood.

Interestingly, a horse can adapt to eating richer food with increased sugar^8^. However, with too much sugar, metabolic syndrome, obesity and laminitis (a dreadful equine disease) occur, and horses may present with hyperinsulinemia, insulin resistance and type 2 diabetes mellitus^12^. By eating richer food and sweet compounds, the horse will present with an upregulation of the sodium/glucose cotransporter SGLT1 and develop an increased capacity to absorb sugar^8^. The same happens to humans^20^. The sugar-absorbing capacity may be trainable.

### Diets, digestive systems and lifestyles

#### Lessons from living and extinct vertebrates

As a general rule, animals make large efforts to obtain foods rich in proteins and calories. The greater the energy spent, the larger must be the reward. This concept is very obvious to lions, whose efforts are immense, as their diets are the richest in nature.

A rich diet means little or no fiber, high calories, and high protein. If an animal has the privilege of having this diet, smaller amounts are needed and less frequently. This diet is easier to digest. Small digestive tracts are ideal, as they are sufficient to digest and absorb this “easy meal”, and the digestive system is light to carry, as hunting is necessary.

A very poor diet, such as rough forage, is not disputed; it is abundant, sparing the animal from efforts to obtain it. However, this diet demands very voluminous meals, hard work and good strategies to digest: we are back to the cow. A large and long digestive tube is necessary. This is an undisputable fact^30^.

This idea explains why the largest dinosaurs were herbivores. A bat with a large abdomen is herbivore. The bats with a very small abdomen are vampires (rich food with no fiber). Birds rarely live on vegetable diets because large stomachs and intestines would impede them from flying. Indeed, the richer the diet is, the smaller the ideal digestive tract^30^ ^,^ ^27^. From fish to mammals, this is a valid rule.

Some small rodents are too small to eat poor diets, which would be full of fiber that their small digestive tract cannot process. Nature and evolution select the best answers. Small rodents may evacuate what is incompletely digested with some enzymes (cecotrophy) and then eat their own feces once more (coprophagy)^30^. A solution for a small digestive tract might be to eat the same meal twice to enable extraction of the most from a poor and rough diet.

Primates are not exceptions. Among primates, there are detailed studies of the relationships between the diet and gut morphology. Two externally very similar monkeys, the howler and spider monkeys, have very different sizes and shapes of the digestive tract. Howler monkeys have longer digestive tracts, and they are able to extract most of their nutrients from leaves, while spider monkeys are less efficient at extracting energy from the fiber in their diet^18^. The latter need to search for ripe fruits, highly digestible and rich in energy, without so much fiber.

**FIGURE 1:**
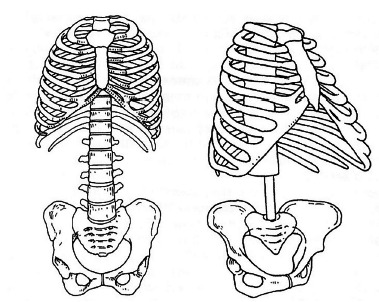
The human abdominal cavity became smaller (left), when compared to primitive *Australopithecus afarensis* (right, reconstructed by Schmid, 1983 apud ref. 1)

In the case of hominids, our ancestors were herbivores 2.5 million years ago. Five Pleistocene glacial/interglacial cycles were recorded in deep-sea sediments over the past 500 thousand years (although only two classic interglacials are recognized on land). These extremely cold periods probably were the main evolutionary pressure that made hominids modify their diet and strategies to obtain it. The scarcity of greens imposed the addition of animal foods; the diet was progressively modified and the digestive system adapted^1^ with a significant diminution being the most visible change (Figure 1).

It is recognized that this change in the diet made insulin resistance an advantageous trait. Indeed, an increase in protein in the diet induces insulin resistance^31^. Populations that were subjected to the selective pressure of very low carbohydrate intake could therefore easily develop hyperglycemia when exposed to high glycemic index foods.

### Scarcity and stomach sizes

Obviously, the rate of gastric emptying^29^ must be limited by the capacity of the intestine to process and absorb food. Indeed, enterohormones (mainly GLP-1) produced by the distal gut reduce gastric emptying very efficiently^19^. In this way, the alimentation process is biphasic: initially, there is hunger, and food is rapidly sent to the intestines, where it is mainly absorbed.After a certain amount is eaten, distal gut signals block the gastric emptying and generate a satiety sensation (an “intestinal satiety”^25^), but animals do not stop eating immediately. Animals have an instinct: gluttony. If there is space in the stomach, they continue eating, even if these nutrients cannot be sent to the intestines immediately. These amounts retained in the stomach represent a stock that will be sent later to intestines. The more intense the scarcity is, more valuable is a large gastric chamber.

It is interesting that this blockage in gastric emptying will provoke a restriction. However, this functional restriction appears only at the end of a meal^29^. Mechanical restrictions (such as gastric bands and rings) that were envisioned for bariatric surgery are permanent and present from the beginning of meals. Mechanical restriction is an obstacle to the ingestion and, obviously, not physiological. These restrictions rather represent a stenosis. Therefore, there is a fundamental difference between a functional and a mechanical restriction. The latter limits ingestion, which is deeply undesirable; the former limits stocking. A good stocking capacity is valuable in scarcity. However, an early functional restriction may be desirable in the (unexpected) circumstance of abundance.

A very reduced gastric chamber, such as the 30 ml pouch resulting from gastric bypass, may empty easily and for too much time, making a large meal possible. This situation is sometimes seen after gastric bypass surgery and can be associated with full weight regain, especially after a great hypertrophy of the proximal gut limb adapts the gut to this extra work^5^.

Therefore, the size of gastric chambers is not linearly related to meal size. It is related to the amount of food that can be stored when gastric emptying is blocked by the “intestinal satiety”. If the intestinal capacity of absorption is great, nutrients are rapidly absorbed, and distal gut signals appear late; so,it is possible to eat large amounts independent of the size of the stomach.

There is another interesting reflection on gluttony. Hunger is a sensation that makes you move, spend energy and take risks in search of food. After intestinal satiety, the capacity to eat more is limited, and above all, there is no urgency. Therefore, animals eat for gluttony the food that is easily reachable at no risk or energy expense. In wild conditions, it is a precious opportunity that justifies the existence of this instinct. A lion that just ate will not run after another prey, and its capacity for food digestion is low for some time. However, if fresh meat is available at no effort, the lion will eat some more.

Humans who stay by the table after dinner seem to do exactly the same. They will not move to the kitchen in search of more, but the food that is within reach might be slowly consumed. Hunger and gluttony are terms that lay people use, but these terms might be physiologically defined.

In the exceptional circumstance of extreme abundance, with continuous exposure to high-calorie and inexpensive meals, gluttony and large stomachs may easily cause overnutrition, a hazardous condition.

The “sleeve” gastrectomy: an evolutionary surgical procedure?

In modern times, a proportional form of stomach reduction was proposed, transforming the large pouch into a simple tube: the “sleeve” gastrectomy. It does not cause any difficulty in the passage of food^14^. Indeed, the stomach in this tubular shape has a smaller ability to distend and increase its volume with similar pressures (low compliance); it empties faster, and when gastric emptying is blocked by the action of gut signals^19^, it has a very small storing capacity.

Civilization, in some areas, brought a very unusual circumstance for an animal: a long-term abundance of high-calorie food. In this situation, the gluttony instinct becomes very inadequate as chronic overeating induces obesity, insulin resistance, and an excess of lipids and sugar in the blood. Metabolic syndrome became a greater problem than undernutrition inthese areas.

**FIGURE 2:**
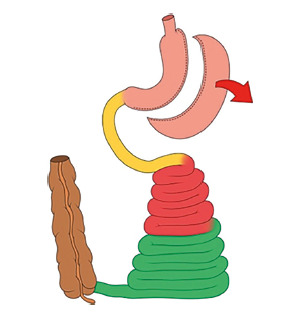
The vertical “sleeve” gastrectomy

The observation of evolutionary steps towards richer food shows that the adaptation to this situation involves smaller stomachs and shorter guts.

As expected, the “sleeve” gastrectomy shows wonderful results in reducing overeating, insulin resistance, diabetes, arterial hypertension, etc.^14^ ^,^ ^15^ in short, the metabolic syndrome. The stomach was not sick or abnormal before, but the “sleeve” gastrectomy represents an advantageous modification in the anatomy and physiology: it is evolution.

However, this modification did not happen by a chance genetic mutation as an ordinary form of evolution. It is a surgically induced phenotypic evolution. Obviously, this new trait will not be transmitted, but it adds benefit to the life of one individual and may be applied to many people who are susceptible to overeating, which is an outstanding achievement.

### The “evolutionary surgery” of the small gut

Today, we have full access to sugar in an amount and concentrations that are not found in nature^4^. This has been a sudden change. Our anatomy was not developed for it. Our proximal gut is still very much dedicated to gluconeogenesis. It is not a surprise that a drug (metformin) which blocks gluconeogenesis would become so popular^16^.

Following this rationale, in a civilized environment of abundance of high-calorie and highly absorbable food (generating a high glycemic index), distal gut signals are proportionally attenuated compared to the proximal gut. Distal gut signals promote the lowering of blood sugars and lipids, satiety and a blockage of gastric emptying and may avoid overeating. Additionally, the hyperplasia that is produced in the proximal gut by overeatingfurther enhances the absorptive capacity^20^, as mentioned earlier. Less distal gut signals imply eating more, in a vicious circle^26^.

Not surprisingly, all the intestinal surgical procedures that work metabolically entail a diminution of the exposure of nutrients to the proximal gut and/or an early contact with the distal gut. Additionally, the actions in this proximal-distal balance are very much independent.

There is metabolic benefit by diminishing proximal gut activity, such as the use of duodenal-jejunal bypass sleeve devices, which create an impermeable barrier between nutrients exiting the stomach and the mucosa of the duodenum and the very proximal jejunum^13^.

**FIGURE 3:**
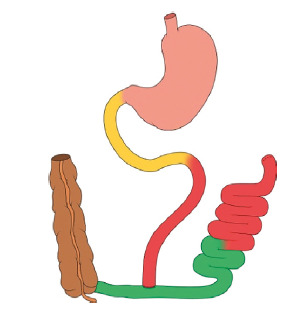
The jejuno-ileal bypass

On the other hand, in jejunum-ileal bypass surgeries performed in the past, the chyme was sent from the proximal jejunum straight to the distal ileum (Figure 3), without any procedure that affected the stomach or the duodenum. There are immediate positive metabolic results as well. The insulin requirements were dramatically reduced following the procedure, and this occurred early in the immediate postsurgical period prior to any significant weight reduction^21^. Both diminishing proximal gut exposure to nutrients and enhancing the exposure of the distal gut work well to improve metabolic results.

The observation from different species shows that natural selection induces a reduction in the small gut as an answer to progressively richer diets with less fiber. The ileum comes closer^30^.

Among all these intestinal procedures, the only one that could be called an “evolutionary procedure”, as the gastric reduction of sleeve gastrectomy would be a proximal bowel reduction - an enterectomy, as it indeed reduces the gut and keeps it aligned (smaller but with the same general design, same sequence of epithelia with no exclusions). This is what is observed in nature as a response to richer diets^27^.

There are animals with different small bowel length, but there are no animals with bypasses, interpositions or bipartitions. However, the sleeve gastrectomy with an enterectomy still might not be the best surgical alternative, in spite of the support of natural observations.

A critical analysis of the procedures is needed to determine what is simpler and what gives the best adaptation to the new environment with fewer complications. Nonetheless, it is clear that the best solutions must reduce the proximal bowel activity and enhance the distal bowel activity.

## DISCUSSION

### Brief critical analyses of metabolic intestinal procedure methods

Four different methods of interfering in the gut anatomy can be used with metabolic purposes: exclusion, transposition, bipartition and resection.

Exclusions of a segment became popular in gastric bypasses (short exclusions) and biliopancreatic derivations (BPDs, long exclusions). They brought positive results after a final balance between pros and cons, and they are widely used. Gastric bypass has become a very popular procedure.

Nonetheless, exclusions were not designed by surgeons thinking either of the complex metabolic roles of the proximal gut or the evolutionary history of the small guts. These procedures were designed to obtain caloric malabsorption; however, it is hard to obtain. If the procedure is so aggressive that it indeed is achieved (like the BPDs), the resulting malnutrition is pronounced and hard to overcome.

Short segments of exclusion cause minimal to no caloric malabsorption. Gastric bypass with average excluded limbs of approximately 0.5 to 1 m work because of the complex and positive interference in metabolic gut signals, partially deactivating the proximal gut and enhancing and accelerating distal gut signals, which is what we generically call the proximal-distal imbalance^25^.

The fine mechanisms behind the correction of this imbalance are gradually being discovered, and they involve gut hormones, bile salt metabolism, changes in microbiota, patterns of gastric emptying, food preferences, energy expenditure, transintestinal cholesterol excretion, and brown fat.

Exclusions have downsides. Even if not producing malabsorption of sugar and fat, non-caloric malabsorption is frequently present and may cause disease. If the exclusion is short, the adaptive mucosal hypertrophy may overcome and compensate for the excluded segment. The patient may, after a few years, regain most of the weight, and the comorbidities may return. The surgical benefit may vanish with time, and gastric bypasses are difficult to revise surgically.

If the exclusion is long, as in BPDs, the results are very superior. However, the malabsorptive events are severe.

Moreover, the exclusion method has other drawbacks, such as dumping syndrome and a lack of endoscopy access. If malabsorption is no longer the goal, the exclusion as a method may possibly be dismissed.

The ileal transposition^17^ allows all the food to pass through the duodenum. The duodenum is the most active part of the proximal gut, and procedures that allow full passage have less potent results, which led the authors of these modalities to include a duodenal exclusion^11^ ^,^ ^10^. In this later form, the procedure is very effective but highly complex, with many mesentery defects and many possibilities for later complications.

**FIGURE 4:**
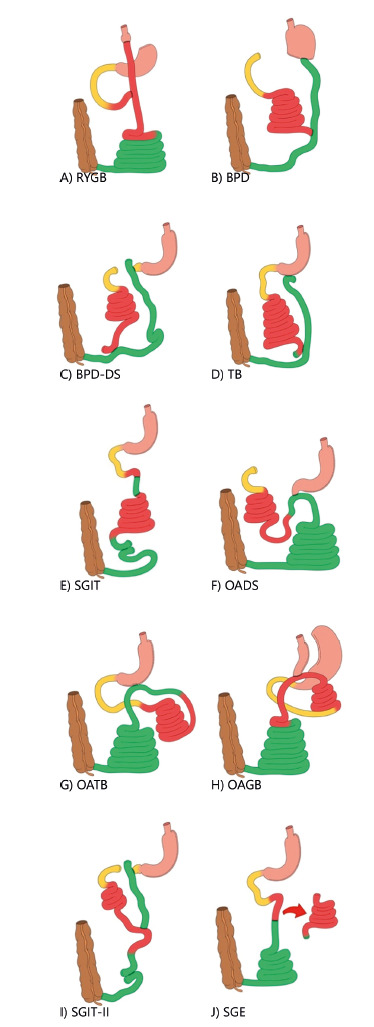
The classic and other bariatric/metabolic surgical models (different from just sleeve gastrectomy), from top to bottom, and from left to right: A) Roux-en-Y gastric bypass (RYGB); B) distal gastrectomy with biliopancreatic derivation (classic BPD); C) SG with duodenal switch (known as BPD-DS); D) SG with transit bipartition (TB); E) SG with ileal transposition (SGIT); F) SG with one-anastomosis duodenal switch (OADS, also known as SADI-S); G) one-anastomosis transit bipartition (OATB, also known as Loop Bipartition and SASI); H) one-anastomosis gastric bypass (OAGB, also known as mini-gastric bypass MGB); I) SG with ileal transposition and duodenal exclusion (known as SGIT-II); J) SG with enterectomy (SGE).

Adding a proximal bowel resection to a sleeve gastrectomy^28^ mimics the steps of evolution: a proportional miniaturization of the gastrointestinal tract. Small bowel resectioning is simple and even safer than the sleeve gastrectomy itself: the anastomosis is safe; the small bowel presents a large variation in normality and a great capacity for adaptation (compensatory hypertrophy) by enhancing distal gut signals (one of the goals). In theory, this procedure would remove portions that are “currently excessive”. The remaining gut may be as long as the lower limit of normality, thereby taking advantage of the immense anatomical variation among humans. The result is an aligned digestive tract with all its portions working well. However, by this method (Figure 4), all high glycemic index food still passes through the duodenum, the major site of simple sugar absorption, the ileum is still not as close as in a BPD and there is the inconvenience of the resection (the loss of reserve).

In this scenario, the transit bipartition brings the ileum up to the antrum, creating rapid ileal stimulation as in a BPD. The whole proximal gut is partially deactivated by the shift of food through the gastroileal anastomosis. As malabsorption is not the goal, but rather a new balance in the proximal and distal gut activities, exclusion is exchanged by the bipartition. Endoscopic access is maintained. The intestinal procedure helps to diminish the high intragastric pressure of the sleeves, and indeed, it utilizes this high pressure as a motor for propelling food through the gastroileal anastomosis.

Both retrospective^24^ and prospective^3^ data show a potency that is similar to the published results for BPDs, and therefore, these approaches seem to be superior to sleeve and gastric bypasses.

In spite of the wide array of effective surgical alternatives, evolutionary biology helps to understand the metabolic surgery in general. Indeed, providing glucose and insulin together is the core activity of the proximal gut. In excess, this situation may result in insulin resistance and obesity. The distal gut, on the other hand, is responsible for lowering blood glucose (potentiation of insulin secretion with blockage of glucagon secretion), diminishing gastric emptying and satiety. The deficiency in these activities may result in the same conditions. All effective metabolic surgery procedures manage to reduce proximal gut activities and enhance distal gut activities

## CONCLUSION

There have been two abrupt and major changes in the human diet: the abundance and the refinement of food (a form of enrichment and pre-digestion). Unnatural elements, such as refined sugar, refined flour and other predigested nutrients were added in large amounts. Changes in the quality and amounts of foods require modifications in the digestive tract.Natural evolution is slow and cannot help a single existing being. It just can improve future populations using the means of discrimination of genes: early death, sexual repugnance and sterility. Surgery that resembles evolutionary movements with physiological support may be a new therapeutic path for a class of diseases that represent a lack of adaption rather than ill organs. Indeed, current models of effective metabolic surgery seem to respect the evolutionary demands.
